# Supplementary material for: Trace elements in fish species from the Punjnad headworks: Bioaccumulation and human health risk assessment
Source: PLoS One. 2025 Jan 24;20(1):e0310744. doi: 10.1371/journal.pone.0310744 (PMC11761173; doi:10.1371/journal.pone.0310744)
Supplement: S1 Table — (DOCX) [file pone.0310744.s001.docx]

**Table S1.** Average weight, Fork, and Total lengths of fish sampled from the study sites at LB and RB of Punjnad. headworks

| **Fish Species** |  | **Average weight (g)** | | | **Average Fork Length (cm)** | | | **Average Total Lengths (cm)** | | |
| --- | --- | --- | --- | --- | --- | --- | --- | --- | --- | --- |
|  |  | **Winter** | **Spring** | **Summer** | **Winter** | **Spring** | **Summer** | **Winter** | **Spring** | **Summer** |
| ***C. mrigala*** | **RB** | 270.90 | 256.42 | 261.49 | 69.24 | 42.85 | 53.59 | 74.92 | 50.75 | 60.27 |
|  | **LB** | 281.02 | 280.74 | 262.94 | 83.59 | 83.39 | 72.01 | 117.63 | 90.63 | 80.29 |
| ***W. attu*** | **RB** | 263.92 | 277.44 | 270.87 | 108.13 | 147.14 | 131.37 | 115.82 | 154.84 | 138.91 |
|  | **LB** | 195.82 | 210.33 | 208.22 | 54.97 | 64.85 | 64.39 | 62.71 | 72.49 | 71.17 |
| ***R. rita*** | **RB** | 208.10 | 226.18 | 223.37 | 95.3 | 115.09 | 110.85 | 102.82 | 122.73 | 118.54 |
|  | **LB** | 181.13 | 185.55 | 194.83 | 69.65 | 74.24 | 76 | 77.17 | 82.07 | 83.97 |
| ***M. armatus*** | **RB** | 274.67 | 283.89 | 291.99 | 84.86 | 97.18 | 107.8 | 104.39 | 104.83 | 115.47 |
|  | **LB** | 328.68 | 329.05 | 320.99 | 130.73 | 136.93 | 116.1 | 135.74 | 142.37 | 121.18 |
| ***S. sarwari*** | **RB** | 291.77 | 295.72 | 287.26 | 101.8 | 110.72 | 87.05 | 119.71 | 128.55 | 104.8 |
|  | **LB** | 475.65 | 394.60 | 352.65 | 148.74 | 130.94 | 122.73 | 166.45 | 148.72 | 141.45 |

**† RB = right bank of Punjnad headworks; LB = left bank of Punjnad headworks**
